# Integrated analysis of microRNA and mRNA expression profiles in splenomegaly induced by non-cirrhotic portal hypertension in rats

**DOI:** 10.1038/s41598-018-36297-0

**Published:** 2018-12-20

**Authors:** Junji Saruwatari, Chao Dong, Teruo Utsumi, Masatake Tanaka, Matthew McConnell, Yasuko Iwakiri

**Affiliations:** 1 Section of Digestive Diseases, Yale University School of Medicine, New Haven, CT USA; 2 Department of General Surgery, Xiangya Hospital, Central South University, Changsha, China; 3 VA CT Healthcare System, West Haven, CT USA

## Abstract

The spleen plays an important role in the immune and hematopoietic systems. Splenomegaly is a frequent consequence of portal hypertension, but the underlying molecular and cellular mechanisms remain to be fully elucidated. In this study, we have performed a whole-genome microarray analysis combined with histological examination in enlarged spleens isolated from rats with partial portal vein ligation (PPVL) surgery to provide comprehensive profiles of microRNAs and their target mRNAs with a focus on their potential biological functions. A total of 964 mRNAs and 30 microRNAs showed significant differential expression in the spleens of PPVL rats compared to rats undergoing a sham procedure. Twenty-two down-regulated microRNAs were associated with significantly increased genes highly involved in fibrogenic activity and cell proliferation/migration (e.g., *Ctgf*, *Serpine1*, *Col1a1*). Consistently, histological analyses demonstrated increased splenic fibrosis and cell proliferation in the spleens of PPVL rats. Eight up-regulated microRNAs were associated with suppression of genes that are related to interferon-mediated antiviral activity in innate immune responses (e.g., *Irf7*, *Dhx58*). In conclusion, we determined a specific microRNA-mRNA network potentially implicated in the tissue fibrosis and cell proliferation in portal hypertension-induced splenomegaly. Our findings provide new insight into the mechanisms for regulation of spleen structure and function.

## Introduction

The spleen is an important organ for the immune system, generating antibodies, removing microbes, and serving as a reservoir for immune cells^1,2^. It also modulates blood components, providing red blood cells when they are deficient during hemorrhagic shock, recycling iron, and capturing circulating platelets^1,2^. Currently, the regulation of spleen structure and function remains to be fully elucidated. Profiling of transcriptomes in the spleen will, therefore, provide novel insights into the regulation of the structure and function.

Splenomegaly is a consequence and an important clinical indicator of portal hypertension^3^. Guidelines addressing portal hypertensive bleeding in cirrhosis recommend routine radiographic assessment of spleen size as an aid to clinical decision-making^4^, and spleen stiffness is increasingly recognized as an important diagnostic tool in the assessment of patients with chronic liver disease^5^. The role of the spleen as active player or passive bystander in portal hypertension, however, remains controversial. Some clinical studies have indicated improvement in portal hypertension following splenectomy in patients^6^. These studies suggest that splenomegaly is an important potential therapeutic target in portal hypertension; however the molecular mechanisms of portal hypertensive splenomegaly remain poorly understood^7,8^. Elucidating these mechanisms may lead to therapeutic options for patients with portal hypertension targeting the spleen that do not carry the substantial risks of surgery.

MicroRNAs (miRNAs) are a class of non-coding RNA molecules that play a key role in a wide range of biological processes, including cell differentiation, proliferation and survival, by binding to complementary target mRNAs, resulting in mRNA translational inhibition or degradation^9,10^. miRNAs have also emerged as critical regulators in the development of immune and inflammatory responses^10–12^. Identifying miRNAs and their target mRNAs on a genome-wide scale could thus provide valuable information to understand the mechanisms of splenomegaly and gain insight into its pathological consequences.

The study presented here describes the use of whole-genome microarray analysis combined with histological analysis to provide a comprehensive profile of miRNAs and their target mRNAs linked to pathophysiological changes associated with splenomegaly in rats who have undergone partial portal vein ligation (PPVL) surgery. PPVL, representing non-cirrhotic portal hypertension, is one of the most frequently used experimental models of portal hypertension, allowing us to examine the mechanisms by whcih increased portal venous pressure alone, without other confounding factors, can lead to splenomegaly^13^. The results from the current study reveal a miRNA-mRNA network that defines structural and biological alterations that occur in portal hypertension-induced splenomegaly.

## Results

### Transcriptional profile of mRNAs in enlarged spleens of rats with portal hypertension – Filtering of microarray data

**Figure 1:**
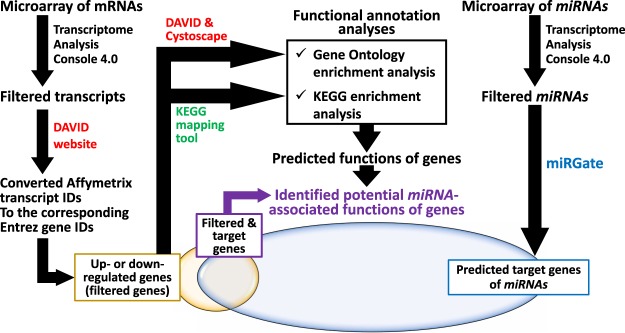
The workflow of mRNA and miRNA data analyses.

**Figure 2:**
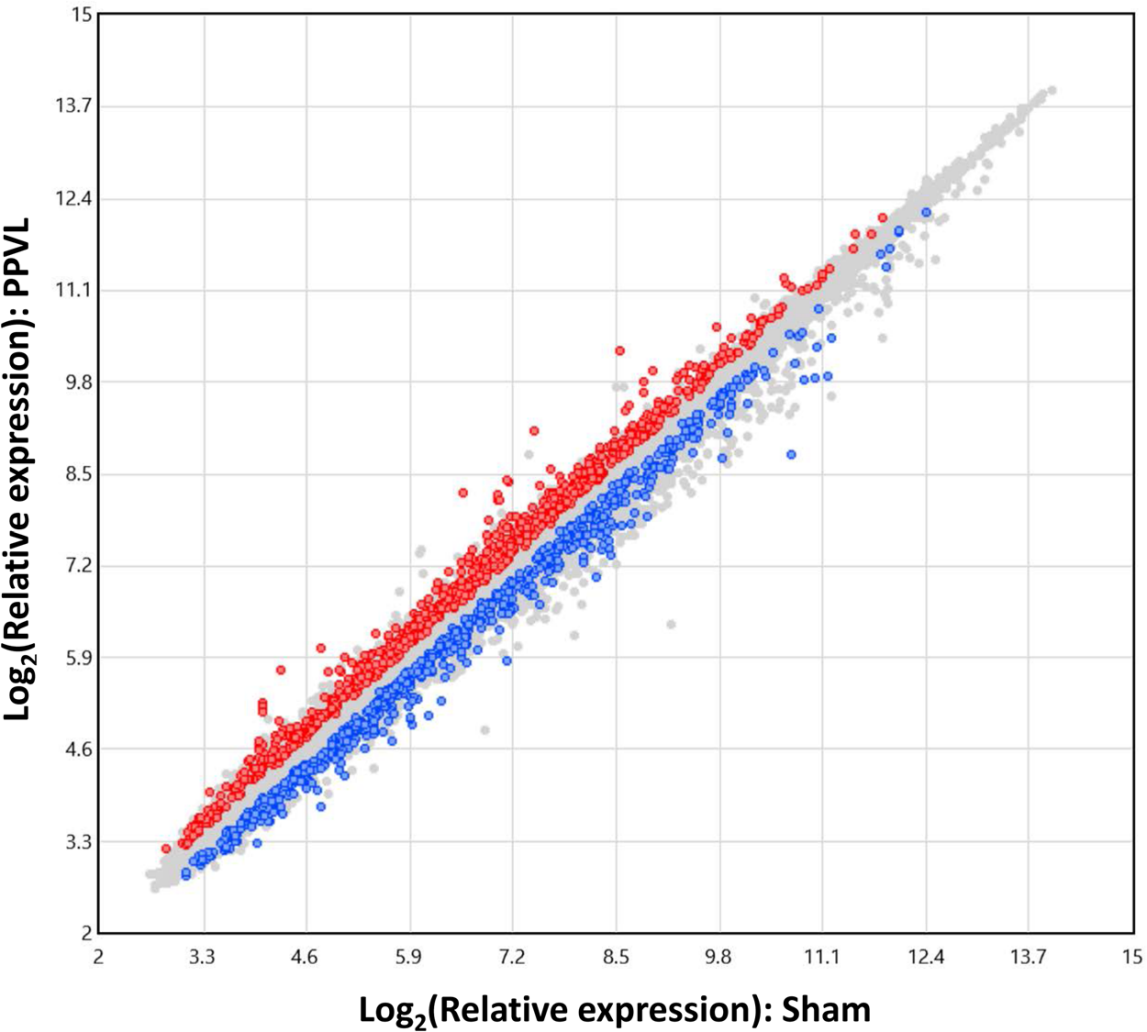
Scatter plot of differentially expressed genes. The plot is color-coded using red for up-regulated genes and blue for down-regulated genes.

A flowchart of mRNA and miRNA data analyses for enlarged spleens of rats with portal hypertension and control spleens is presented in Fig. 1. Expression of 1,549 transcripts out of 28,407 genes differed on the microarray between these two groups (Fig. 2). Among them, 964 genes (Supplementary Fig. S1), of which 599 genes were up-regulated and 365 genes were down-regulated in the spleens of PPVL rats, were recorded as protein coding genes in Entrez Gene, the gene-specific database of the National Center for Biotechnology Information^14^. Forty out of the 599 up-regulated genes showed a greater than 1.5-fold increase with 10 of them increasing by more than 2-fold, while 41 out of the 365 genes were down-regulated by more than 1.5-fold with 8 of them decreasing by more than 2-fold. Table 1 lists the top 15 up- and down-regulated genes in the order of fold-change.

**Table 1:** List of top 15 genes up- or down-regulated in the spleens of PPVL rats compared to those of sham rats.

Entrez Gene ID	Gene Symbol	Gene Description	Average log2 value		Fold-change	*P*-value^a^
			Sham	PPVL		
***Up-regulated***						
286995	Defa5	defensin, alpha 5, Paneth cell-specific	8.55	10.24	**3.23**	0.0012
286958	Np4	defensin NP-4 precursor	6.57	8.24	**3.18**	0.0016
498659	RatNP-3b	defensin RatNP-3 precursor	7.46	9.12	**3.15**	0.0009
613225	Defa11	defensin alpha 11	4.27	5.71	**2.72**	0.0172
314322	Fos	FBJ osteosarcoma oncogene	7.11	8.41	**2.45**	0.0003
58826	Prg2	proteoglycan 2	7.14	8.4	**2.4**	0.0287
305941	Sgcg	sarcoglycan, gamma	4.77	6.02	**2.38**	0.0002
81686	Mmp2	matrix metallopeptidase 2	7.01	8.21	**2.29**	0.0101
29266	Mcpt2	mast cell protease 2	4.04	5.22	**2.25**	0.0002
54289	Rgs1	regulator of G-protein signaling 1	7	8.12	**2.17**	0.0061
29393	Col1a1	collagen, type I, alpha 1	8.85	9.81	**1.95**	0.0068
24617	Serpine1	serpin peptidase inhibitor, clade E (nexin, plasminogen activator inhibitor type 1), member 1	6.9	7.85	**1.93**	8.10E-06
25043	Eln	Elastin	7.68	8.57	**1.85**	0.0167
287382	Mfap4	microfibrillar-associated protein 4	8.83	9.66	**1.77**	0.0141
282836	Cthrc1	collagen triple helix repeat containing 1	5.08	5.9	**1.76**	0.0105
***Down-regulated***						
363938	Oas2	2–5 oligoadenylate synthetase 2	8.34	7.5	**−1.78**	0.001
361042	Pck2	phosphoenolpyruvate carboxykinase 2 (mitochondrial)	8.41	7.55	**−1.81**	0.0333
297666	Klra5	killer cell lectin-like receptor, subfamily A, member 5	5.09	4.23	**−1.81**	0.0109
309526	Ifit3	Interferon-induced protein with tetratricopeptide repeats 3	8.31	7.43	**−1.84**	6.26E-05
295704	Ube2l6	ubiquitin-conjugating enzyme E2L 6	8.69	7.79	**−1.87**	0.0042
303538	Dhx58	DEXH (Asp-Glu-X-His) box polypeptide 58	8.42	7.46	**−1.94**	7.24E-05
311546	Tpx2	TPX2, microtubule-associated	8.89	7.9	**−1.99**	0.0483
619560	Rup2	urinary protein 2	4.79	3.78	**−2.01**	0.0293
303175	Hist3h2ba	histone cluster 3, H2ba	6.3	5.28	**−2.03**	0.0195
293624	Irf7	Interferon regulatory factor 7	10.86	9.84	**−2.04**	0.0025
246268	Oas1b	2–5 oligoadenylate synthetase 1B	8.43	7.35	**−2.11**	5.43E-05
414788	RT1-T24-3	RT1 class I, locus T24, gene 3	9.82	8.72	**−2.14**	0.0092
114247	Slfn4	schlafen 4	11	9.85	**−2.22**	5.41E-05
298693	Isg15	ISG15 ubiquitin-like modifier	8.24	7.05	**−2.28**	0.0006
294090	Ifit1bl	interferon-induced protein with tetratricopeptide repeats 1B-like	10.7	8.76	**−3.84**	0.0167

^a^Indicates p-values calculated by an empirical Bayes approach.

### Gene ontology and pathway analyses

To determine biological functions that could be influenced in the spleens of PPVL rats, we performed gene ontology (GO) analysis and categorized the identified genes into biological functions (‘GO terms’) constructed by biological process, molecular function and cellular component^15,16^. An analysis of all genes up- and down-regulated in the spleens of PPVL rats using the Functional Annotation Chart Tool of the Database for Annotation, Visualization and Integrated Discovery (DAVID) (Fig. 1) identified 335 and 65 GO terms for the genes up- and down-regulated, respectively. GO terms most often annotated were “*extracellular space*” for the genes up-regulated and “*cytoplasm*” for the genes down-regulated (Table 2).

**Table 2:** List of GO terms with the largest number of genes up- or down-regulated.

GO No.	GO Term	Fold Enrichment^a^	*P*-value^b^	Gene	
				Fold-change	Gene Name
***Up-regulated***					
GO:0005615	extracellular space	2.13	1.02E-10	>2.0	Defa5, Defa11, Np4, RatNP-3b, Mmp2
				>1.5	Adipoq, Col1a1, Col3a1, Cthrc1, Ctgf, Cpz, Dpep1, Eln, Flrt2, Gpx3, Mpo, Ngp, Serpine1, Sfrp2
				>1.0	Acpp, Alpl, Apln, Bmp2, Bmpr2, Bmp3, Bmp6, C1qtnf1, C1qtnf5, C8g, Ccl2, Cd14, Clec11a, Cfh, Col1a2, Cpxm2, Ctsg, Ctsk, Dpysl3, F8, Fgf1, Fgf2, Figf, Frmd4b, Fstl1, Gas6, Ghr, Glb1, Grem1, Igf1, Il6st, Inhba, Jam3, Lamb1, Lgals3, Lox, Lrig3, Ltbp2, Masp1, Mertk, Nenf, Nrg1, Ogn, Pltp, Prelp, Prtn3, Ptprg, Pxdn, RGD1305645, Sema4g, Sepp1, Serpine2, Serpina10, Serping1, Serpinb1a, Sipa1l3, Slit2, Sost, Sparc, Srpx2, Tgfb3, Try10, Vegfb
***Down-regulated***					
GO:0005737	cytoplasm	1.20	0.02481	>2.0	Irf7
				>1.5	Brca1, Dhx58, Fancd2, Ifit3, Mx1, Mx2, Oasl, Oas2, Orc1, RGD1563091, Tppp3
				>1.0	Aatf, Ablim2, Adam17, Aifm1, Aipl1, Akr1d1, Ankrd2, Apob, Aqp11, Bambi, Ccr5, Cct8, Cdc7, Cdc25c, Cdk2, Cep57, Chn2, Ciapin1, Clic3, Colec10, Dbil5, Dbndd1, Dcx, Ddias, Ddx58, Ddx60, Dnali1, Dsn1, Dtx3l, Elp4, Ercc6l, F13a1, Fam126a, Fscn3, Gadd45b, Gemin2, Grpel2, Gzmb; Gzmbl2, Gzmf, Haus7, Herc4, Herc6, Hist1h2bo, Hspb3, Ifih1, Ifit2, Ifng, Ing2, Irf9, Isg20, Kntc1, Krt27, Mad2l1, Mphosph9, Mre11a, Mstn, Nasp, Nr0b2, Parp9, Parp14, Pfas, Ppa1, Prpf3, Rad54b, RGD1562136, Rrm1, Rsb66, Rtp4, Selp, Slc7a12, Slc30a1, Spdl1, Sprr3, Sra1, Tbpl1, Tcp1, Tdrd7, Tiam2, Trim21, Trim69, Tyms, Ubxn11, Usp11, Usp25, Vrk1, Zbp1, Zc3hav1, Zmym1

^a^Denotes the ratio of the number of genes in the gene set to the expected number in the category based on the human database.

^b^Indicates p-values adjusted by the multiple test Benjamini adjustment.

The underlined genes are predicted targets for up- or down-regulated miRNAs in the spleens of PPVL rats.

#### Genes up-regulated in the spleens of PPVL rats

**Figure 3:**
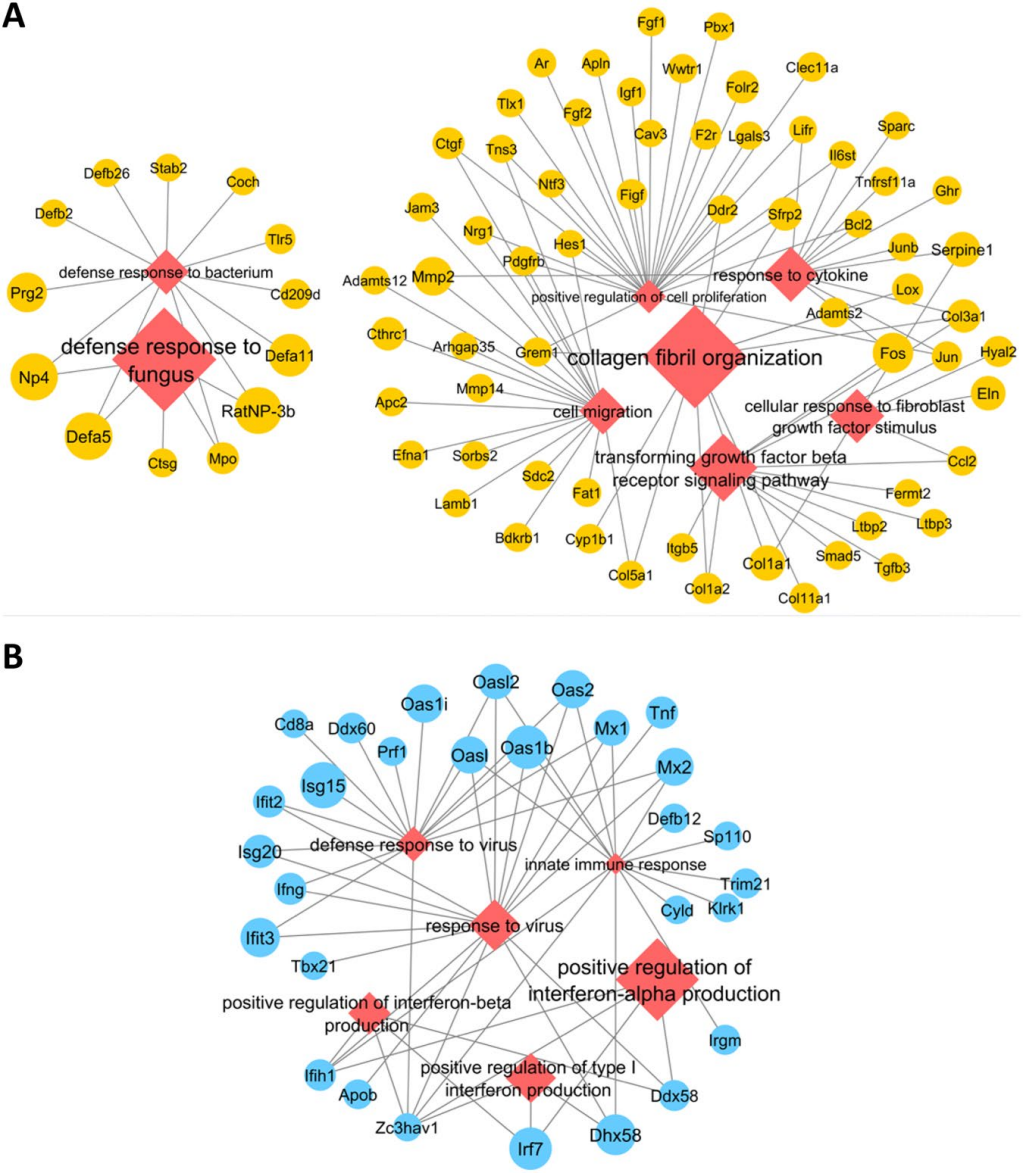
GO terms and their related genes up- or down-regulated in the spleens of PPVL rats. (**a**) The network consists of GO terms (red diamonds) and their related up-regulated genes (yellow circles). (**b**) The network consists of GO terms (red diamonds) and their related down-regulated genes (blue circles). The sizes of circles and diamonds represent the fold-change and the fold-enrichment, respectively, in the spleens of PPVL rats compared to those of sham rats.

Given the results from filtering of microarray data mentioned above, we further charaterized the up-regulated genes associated with the GO term “*extracellular space*” (Table 2) with identification of additional GO terms to which they were also related. Highly up-regulated genes such as α-defensins (*RatNP-3b*, *Np4*, *Defa5* and *Defa11*) were also associated with GO terms “*defense responses to fungus/bacterium*” (Fig. 3A). Genes such as *Mmp2* and *Serpine1* (also known as PAI-1) were related to a GO term “*response to cytokine*” (Fig. 3A). Other up-regulated genes related to “*extracellular space*” were also associated with GO terms such as “*cell migration*”, “*positive regulation of cell proliferation*”, “*cellular response to fibroblast growth factor stimulus*”, “*transforming growth factor beta receptor signaling pathway*” and “*collagen fibril organization*” (Fig. 3A).

**Figure 4:**
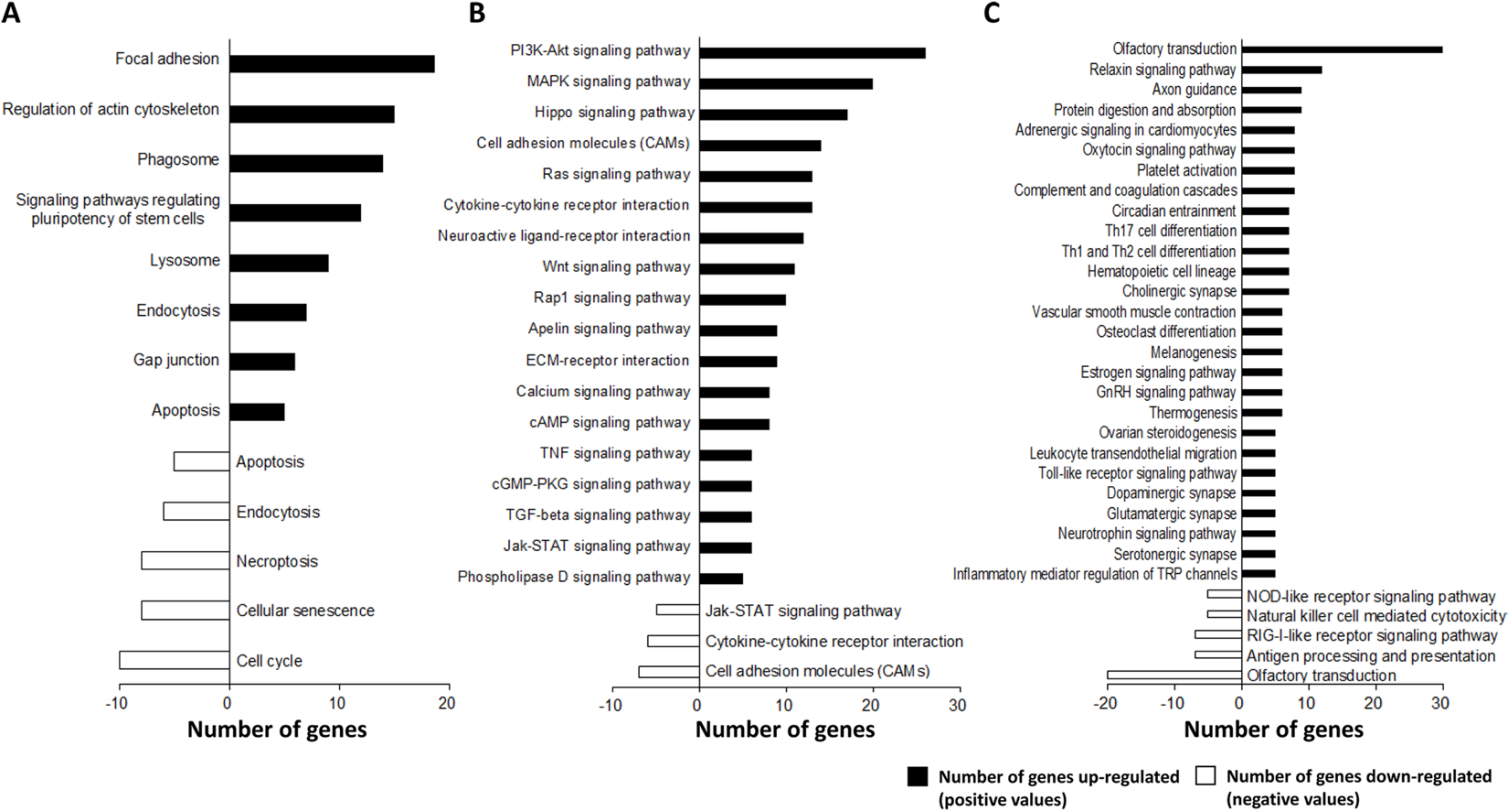
The KEGG pathways with more than five genes up- or down-regulated in three pathway areas: (**A**) “Cellular Processes”, (**B**) “Environmental Information Processing” and (**C**) “Organismal Systems”. The positive and negative numbers represent the number of genes up- and down-regulated, respectively, in the spleens of PPVL rats.

We then performed the Kyoto Encyclopedia of Genes and Genomes Pathway Database (KEGG)-enrichment analysis with a focus on its three areas, “Cellular Processes”, “Environmental Information Processing” and “Organismal Systems”, to explore the main pathways in which these up- and down-regulated genes are involved (Fig. 1). First, we determined specific pathways in the “Cellular Processes” area with the genes up-regulated in the spleens of PPVL rats (Fig. 4A). This resulted in identification of two pathways, “*focal adhesion*” with up-regulated genes such as *Col1a1*, *Col1a2* and *Itga8* and “*regulation of actin cytoskeleton*” with up-regulated genes such as *Myh10*, *Itga8*, *Fgf1/2* and *Pdfgfrb* (Fig. 4A, Supplementary Table S1), suggesting these pathways are possibly activated in the spleens of PPVL rats.

**Figure 5:**
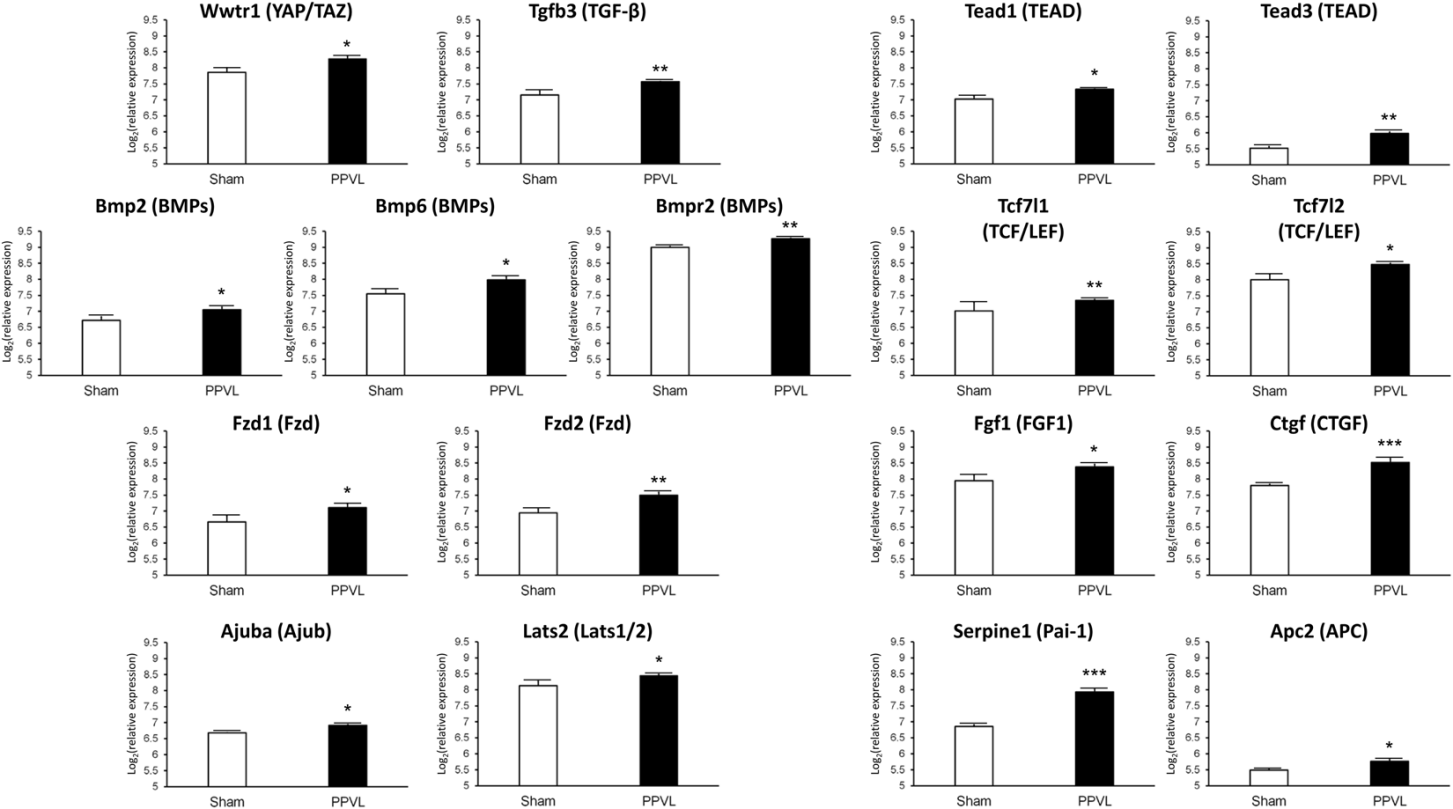
The mean relative expression of genes involved in the KEGG “*Hippo signaling pathway”*. The letters in parenthesis represent the terms cited in the “Hippo signaling pathway” on the KEGG website (http://www.genome.jp/kegg-bin/show_pathway?map04390). “*”, “**” and “***” indicate significance at p < 0.05, p < 0.01 and p < 0.001, respectively, compared to sham group.

Second, we determined pathways in the “Environmental Information Processing” area with the genes up-regulated in the spleens of PPVL rats (Fig. 4B, Supplementary Table S2). The “*phosphatidylinositol 3’-kinase (PI3K)-Akt signaling pathway”* and “*mitogen-activated protein kinase (MAPK) signaling pathway*” were identified with the first and second largest numbers of the genes up-regulated in the spleens of PPVL rats (Fig. 4B). However, the main components of the “*PI3K-Akt signaling pathway*” (i.e., PI3K isoforms and AKT) were not up-regulated (Supplementary Table S2). In the *“MAPK signaling pathway”*, neither the genes of MAPKs (i.e., *ERK*, *JNK* and *p38*) nor those of MAPK kinases (i.e., *MEK1/2*) were up-regulated in PPVL spleens, while enzymes involved in the deactivation of MAPKs (i.e., *Dusp1* and *Dusp7*) were up-regulated (Supplementary Table S2). The “*Hippo signaling pathway*” showed the third largest number of the genes up-regulated in PPVL spleens among the pathways in the “Environmental Information Processing” area (Fig. 4B). In the “*Hippo signaling pathway*”, a transcriptional coactivator *Wwtr1* (also known as TAZ) and its enhancers *Tead1/3* were significantly up-regulated with their target genes, such as *Ctgf*, *Serpine1* and *Fgf1*, up-regulated as well (Fig. 5, Supplementary Table S2), suggesting that TAZ and TEAD could be involved in controlling organ sizes and/or promoting cell growth and proliferation in PPVL-induced splenomegaly.

In the “Organismal Systems” area, we identified “*olfactory transduction*”, “*relaxin signaling pathway*” and “*axon guidance*” with the first, second and third largest numbers of the genes up-regulated in PPVL spleens among the pathways of this classification (Fig. 4C, Supplementary Table S3).

#### Genes down-regulated in the spleens of PPVL rats

Like the case for the up-regulated genes, given that the genes down-regulated were most commonly annotated to the GO term “*cytoplasm*” (Table 2), we looked into additional GO terms for the down-regulated genes related to this term. We found that these down-regulated genes were also associated with GO terms “*positive regulation of type I interferon production*” (including *Irf7* and *Dhx58*), “*positive regulation of interferons*” (including *Irf7* and *Dhx58*), “*innate immune response*” and “*response to virus*” (including *Dhx58*, *Oas1b*, *Oasl*, *Oas2*, *Oasl2*, *Mx1* and *Mx2*) (Fig. 3B).

Based on KEGG-enrichment analysis, we identified the “*cell cycle*”, “*cellular senescence*” and “*necroptosis*” pathways in the “Cellular Processes” area with the first, second and third largest numbers of the genes down-regulated in PPVL spleens (Fig. 4A, Supplementary Table S1). In the “Environmental Information Processing” area, “*cell adhesion molecules (CAMs)*”, “*cytokine-cytokine receptor interaction*” and “*Jak-STAT signaling pathway*” were found with the most substantial numbers of the down-regulated genes, but these pathways were also identified with the genes up-regulated in the spleens of PPVL rats (Fig. 4B, Supplementary Table S2).

In the “Organismal Systems” area, similar to the genes up-regulated, the largest number of the down-regulated genes was annotated to the “*olfactory transduction*” pathway (Fig. 4C, Supplementary Table S3). The “*antigen processing and presentation*” (including *RT1-T24-3*, *Ifng* and *Tfn*) and “*RIG-I-like receptor signaling pathway*” (including *Irf7*, *Isg15* and *Ddx58* also known as LGP2), which are implicated in innate immune responses^17^, followed as the second and third pathways in this area (Fig. 4C, Supplementary Table S3).

### Transcriptional profile of miRNAs and their target genes

To examine the potential involvement of miRNAs in differential gene expression in the spleens of PPVL rats, we performed microarray analysis of miRNAs (Fig. 1). One hundred thirty-one miRNAs (with 39 miRNAs up-regulated and 92 miRNAs *down-regulated*) were differentially expressed out of 1,218 *Rattus norvegicus* miRNAs (including 728 mature and 490 pre-mature miRNAs) on the microarray. Thirty miRNAs with 22 down-regulated and 8 up-regulated showed more than 1.5-fold changes (Table 3).

**Table 3:** List of microRNAs *down-* or *up-regulated* more than 1.5-fold in the spleens of PPVL rats compared to those of sham rats.

microRNA	Average log2 value		Fold-change	*P*-value^a^
	Sham	PPVL		
***Down-regulated***				
rno-miR-3584-3p	3.14	1.44	**−3.26**	0.0115
rno-miR-141-3p	5.26	3.69	**−2.97**	0.0284
rno-let-7i-3p	5.53	4.57	**−1.94**	0.0151
rno-miR-223-5p	2.58	1.63	**−1.93**	0.0195
rno-miR-219b	2.66	1.71	**−1.93**	0.0128
rno-miR-6318	4.01	3.06	**−1.92**	0.0106
rno-miR-147	7.09	6.22	**−1.83**	0.0814
rno-miR-3564	2.48	1.65	**−1.78**	0.0184
rno-miR-34b-3p	6.13	5.31	**−1.76**	0.0183
rno-miR-664-1-5p	7.16	6.35	**−1.75**	0.0273
rno-miR-146b-3p	2.77	2.00	**−1.71**	0.01
rno-miR-1949	9.27	8.51	**−1.69**	0.0627
rno-miR-1224	9.56	8.82	**−1.67**	0.0042
rno-miR-18a-5p	11.26	10.53	**−1.66**	0.08
rno-miR-582-3p	4.36	3.63	**−1.66**	0.0041
rno-miR-551b-3p	2.26	1.53	**−1.66**	0.0452
rno-miR-20b-3p	6.06	5.35	**−1.64**	0.0514
rno-miR-130b-3p	10.86	10.25	**−1.53**	0.0702
rno-miR-1188-3p	2.65	2.04	**−1.53**	0.0693
rno-miR-770-3p	3.79	3.17	**−1.53**	0.0255
rno-miR-409a-3p	6.05	5.45	**−1.52**	0.0481
rno-miR-874-5p	3.07	2.48	**−1.51**	0.0329
***Up-regulated***				
rno-miR-541-5p	6.94	7.56	**1.53**	0.007
rno-miR-193-5p	6.49	7.13	**1.56**	0.0553
rno-miR-125b-1-3p	5.07	5.77	**1.63**	0.0287
rno-miR-411-5p	4.66	5.4	**1.67**	0.0388
rno-miR-351-3p	4.55	5.3	**1.67**	0.0386
rno-miR-31a-5p	8.02	8.97	**1.93**	0.0635
rno-miR-184	5.2	6.32	**2.17**	0.0755
rno-miR-31a-3p	1.98	3.79	**3.51**	0.0344

^a^Indicates p-values calculated by an empirical Bayes approach.

**Figure 6:**
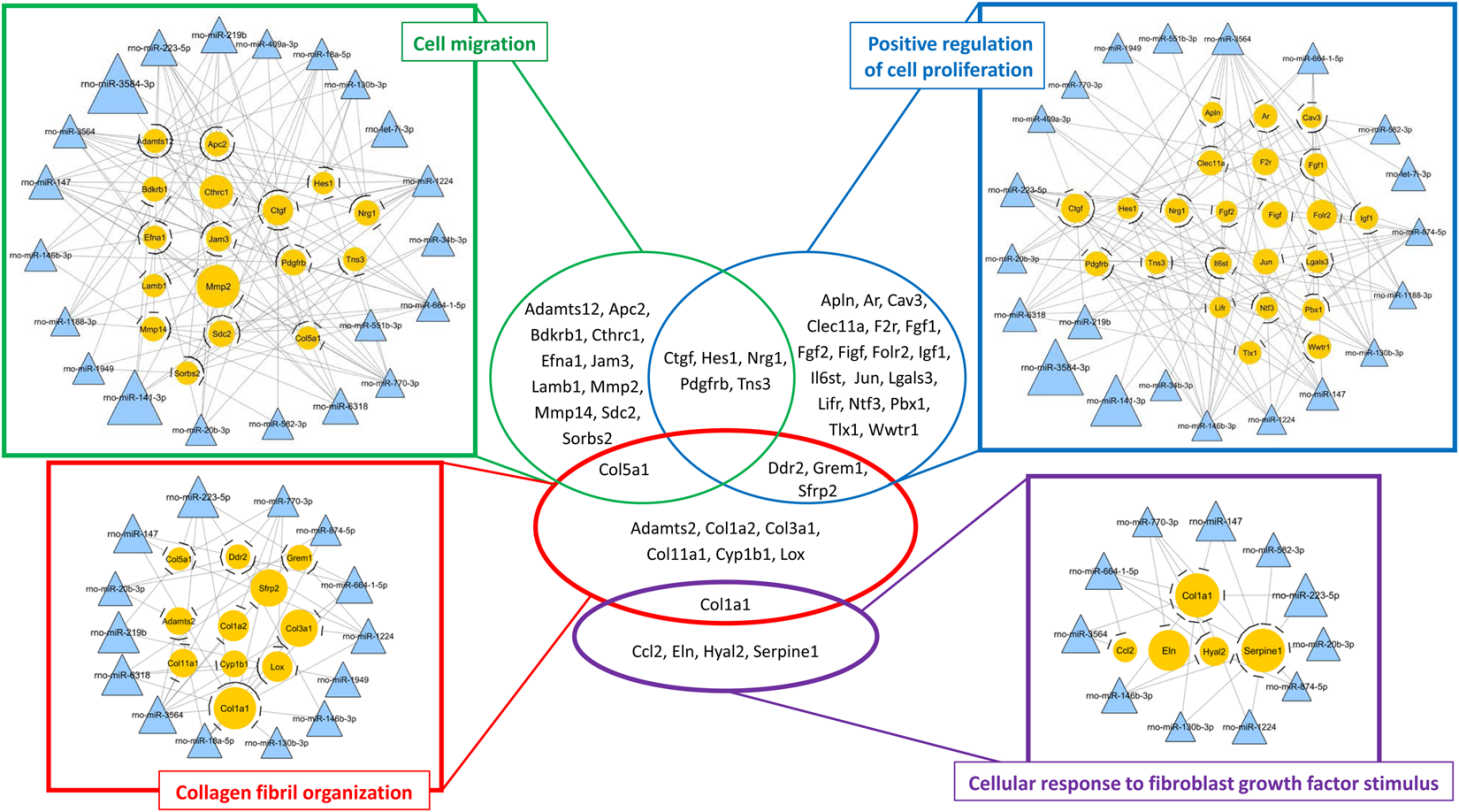
Schematic overview of the specific miRNAs-gene-function relationships identified in the up-regulated mRNA and down-regulated miRNA data analyses in the spleens of PPVL rats. The sizes of circles and triangles represent the fold-change in the spleens of PPVL rats compared to those of sham rats.

We then determined 17,216 genes as predicted target genes of the 22 *down-regulated* miRNAs using the miRGate database. From these predicted target genes, we extracted 484 genes which were up-regulated in the spleens of PPVL rats (Supplementary Table S4). In other words, most (80.8%) of the up-regulated genes in the spleens of PPVL rats were predicted as targets of the 22 down-regulated miRNAs. Consistently, genes related to the GO terms determined above were largely predicted target genes (Table 2). They were involved in *cell migration and proliferation* (*Ctgf*, *Pdgfrb*, *Fgf1/2*, *Mmp2*, etc.) and *cellular response to fibroblast growth factor stimulus/ collagen fibril organization* (*Serpine1*, *Eln*, *Col1a1*, *Col1a2*, *Col3a1*, etc.) (Fig. 6). Likewise, these extracted target genes were associated with KEGG pathways, such as “*focal adhesion*”, “*regulation of actin cytoskeleton*” and *“Hippo signaling pathway”* (Supplementary Tables S1, S2 and S3).

**Figure 7:**
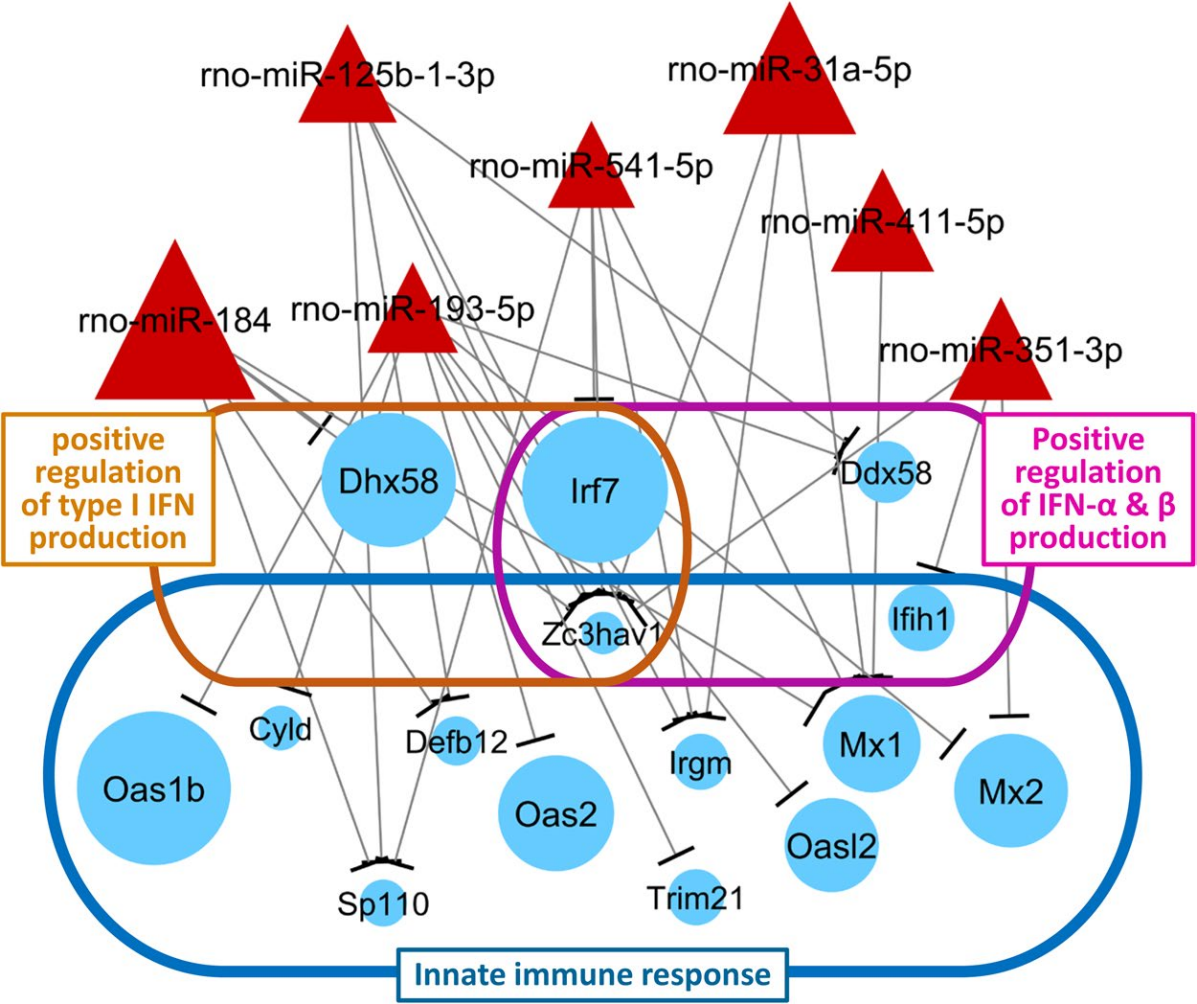
Schematic overview of the specific miRNAs-gene-function relationships identified in the down-regulated mRNA and up-regulated miRNA data analyses in the spleens of PPVL rats. The sizes of circles and triangles represent the fold-change in the spleens of PPVL rats compared to those of sham rats.

Similarly, we determined 13,034 genes as predicted target genes of the eight up-regulated miRNAs and extracted 216 genes which were down-regulated in PPVL spleens (Supplementary Table S5). These extracted genes included genes related to *the production of interferons* (*Irf7* and *Dhx58*) and *innate immune response* (*Oas1b*, *Oas2*, *Mx1* and *Mx2*) (Fig. 7, Supplementary Tables S1, S2 and S3).

Collectively, these results have demonstrated a close connection between our target gene prediction analysis (miRNAs vs. mRNAs) and our functional annotation analysis (mRNAs vs. GO terms and KEGG pathways), providing an integrated network of miRNAs and mRNAs relevant to the pathophysiology of the spleen observed in PPVL rats.

### A significant increase in fibrosis and cell proliferation in the spleens of PPVL rats

**Figure 8:**
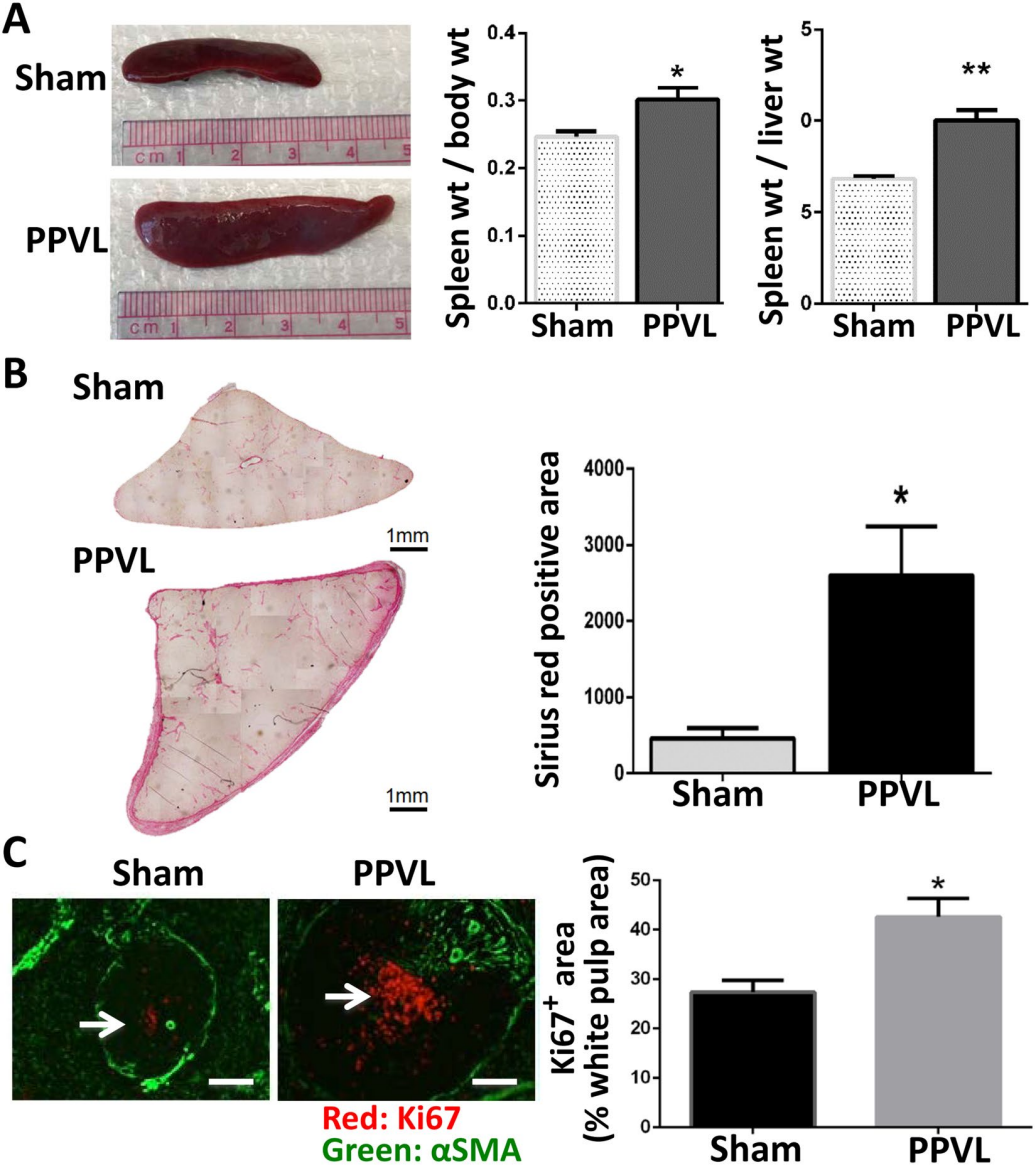
Splenomegaly is associated with increased spleen fibrosis and cell proliferation in rats. (**A**) Comparison of the spleens between sham and PPVL 10-day rats. “*” and “**” indicate significance at p < 0.01 and p < 0.001, respectively. (**B**) Sirius red images of spleens isolated from sham and 10-day PPVL rats. “*” indicates significance at p < 0.05. (**C**) Immunolabeling of Ki67 and alpha-smooth muscle actin (α-SMA). Ki67 was used as a marker for proliferation. Scale bar: 200 μm. Arrows: Ki67 positive cells in the white pulp area. “*” indicates significance at p < 0.01.

Given that a substantial number of the up-regulated genes, most of which are potential targets of the down-regulated miRNAs, were associated with cell proliferation/migration and pro-fibrogenic signaling (Figs 3 and 6), we examined the levels of splenic fibrosis and cell proliferation in our model. Consistent with transcriptional profiles of mRNAs and miRNAs, PPVL rats demonstrated significantly increased spleen weights compared to sham rats (Fig. 8A). Splenomegaly in PPVL rats was associated with significantly increased fibrosis as indicated by Sirius red staining (Fig. 8B). In addition, significantly increased cell proliferation was observed in the spleens of PPVL rats, compared to those of sham rats (Fig. 8C). These observations of increased fibrosis and cell proliferation in enlarged spleens of PPVL rats verify the changes in profiles of miRNAs and corresponding mRNAs found in this study.

## Discussion

This is the first study to determine the integrated miRNA-mRNA network in splenomegaly and its potential roles in the underlying molecular mechanisms and biological changes. The current study has identified unique transcriptome profiles regulating biological processes in splenomegaly induced by PPVL, a model of non-cirrhotic portal hypertension. This study has determined 22 down-regulated miRNAs showing more than 1.5-fold change in the spleens of PPVL rats (Table 3), which included miRNAs that have been implicated in the promotion of fibrosis, cell proliferation and migration (e.g., *miR-141-3p*)^18–20^ and the regulation of inflammatory responses (e.g., *let-7i*, *miR-146b*)^10,11^. Further, our target prediction analysis has identified that among predicted target genes of these 22 down-regulated miRNAs, genes known to be highly involved in fibrogenic activity, cell proliferation and migration (e.g.*, Col1a1*, *Serpine1*, *Ctgf*) were up-regulated in the spleens of PPVL rats (Fig. 6). Importantly, in agreement with these transcriptome profiles, histological analyses have demonstrated increased splenic fibrosis and cell proliferation (Fig. 8). Collectively, our study has identified miRNAs and their target mRNAs, which are potentially involved in splenic fibrosis and cell proliferation observed in PPVL-induced splenomegaly.

This study has also determined 8 up-regulated miRNAs in the spleens of PPVL rats (Table 3), which included miRNAs that have been implicated in the immune response (e.g., *miR-184*, *miR-125*)^21,22^. Among predicted target genes of these up-regulated miRNAs, genes related to IFN-α and β production as well as the innate immune response (e.g.*, Irf7*, *Dhx58*, *Oas1b*, *Mx2*) were down-regulated in PPVL-induced splenomegaly (Fig. 7). In contrast, genes encoding several α-defensins (*RatNP-3b*, *Np4*, *Defa5* and *Defa11*) and *Prg2*, which have potential implications in antimicrobial and antiviral activities *in vivo*^23–25^, were significantly up-regulated (Fig. 3B), but except for *Prg2*, were not associated with the miRNAs down-regulated in the spleens of PPVL rats (Supplementary Table S4). These findings may indicate potential distinctive alterations in defense mechanisms in PPVL-induced splenomegaly with miRNA-dependent interferon-mediated antiviral activities being deactivated, but with miRNA-independent defense response by α-defensins activated. Regarding α-defensins, other subtypes, α-defensin-1 and -2, are also known to induce fibroblast proliferation and fibrosis in liver and lung tissues^26–28^. Utilizing the strength of our unbiased screening approach to discover unexpected connections in biological systems, we have identified an association between up-regulated α-defensins in the spleen and splenic fibrosis. Given the role of α-defensins in fibrosis in other organ systems, this suggests a novel mechanism of portal hypertensive splenic fibrosis warranting further investigation.

Besides portal hypertension, many other pathological conditions such as infection and hematological malignancies can cause splenomegaly^2,13^. Gene expression profiles of splenomegaly may differ according to etiology. Schistosoma infection is a frequent cause of portal hypertension and subsequent splenomegaly in developing countries^29^. A whole-genome microarray analysis was performed recently in enlarged spleens of mice infected with *Schistosoma japonicum*^30^. In comparison with our results, at least three distinct gene expression profiles are noted. First, extracellular matrix (ECM)-related gene expression (e.g., *Ctgf* and *Fgf1*) was significantly decreased in the spleens of Schistosoma-infected mice^30^, whereas these genes were significantly up-regulated in our study (Table 2, Fig. 3A). Second, in contrast with our study (Table 2, Fig. 3B), Schistosoma-induced splenomegaly was related to up-regulation of several interferon-inducible genes such as *Irf7*, *Oas2* and *Ifit2*. Third, Schistosoma-induced splenomegaly showed decreased expression of lymphocyte chemokines (e.g., *Cxcl13*, *Ccl19* and *Ccl21*)^30^, while no changes were observed in our study. These differences in gene expression profiles may indicate different mechanisms underlying splenomegaly according to etiology.

Other studies also showed that splenomegaly was associated with splenic fibrosis in rats with PPVL^31^ and that mTOR signaling promoted splenomegaly in these rats^31,32^. While splenic fibrosis was in agreement with our study, our results do not implicate genes related to mTOR signaling in splenomegaly. In these previous studies, expression of phosphorylated 4E-BP1 and p70-S6K proteins, two direct downstream targets of the mTOR kinase, were used to measure the activity of mTOR^31,32^. The discrepancy between our results and these previous findings may be due to differences between the transcriptional regulation we determined and the posttranslational regulation of downstream mTOR signaling (i.e., phosphorylated states of 4E-BP1 and p70-S6K)^31,32^.

Increased ECM levels may promote splenomegaly, since mechanical signals from the ECM (e.g., stiffness and elasticity) may help splenic cells to proliferate, differentiate and prevent cell death^33^. Mechanical signals induce activation of YAP/TAZ transcriptional coactivators, both of which are downstream transducers of the Hippo signaling pathway and essential effectors of ECM mechanical cues^34^. YAP/TAZ activity is key to the growth of organs, amplification of tissue-specific progenitor cells during tissue renewal and regeneration, and cell proliferation^34^. KEGG enrichment analysis of this study identified a substantial number of up-regulated genes that are involved in the Hippo signaling pathway, including TAZ, TEAD (a key DNA-binding platform for YAP/TAZ) and their downstream genes such as *Ctgf*, *Serpine1* and *Fgf1* (Fig. 5, Supplementary Table S2). These downstream genes are known to play substantial roles in many cellular responses and pathological processes including cell adhesion, proliferation, migration, and tissue fibrosis^34–37^. Further studies linking the Hippo signaling pathway and YAP/TAZ activation to increased splenic fibrosis and cell proliferation may provide a novel mechanistic insight into PPVL-induced splenomegaly.

One previous study using microarray determined gene expression profiles in blood samples from patients with idiopathic portal hypertension^38^. The study showed up-regulation of genes that are involved in the actin cytoskeleton, actin binding and cytoskeletal protein binding^38^ as well as down-regulation of genes primarily related to the immune system, suggesting immunological abnormalities in idiopathic portal hypertension^38^. Similarly, our functional annotation analyses indicated increased actin cytoskeleton (Fig. 4A) and immunological abnormalities (Fig. 3) in the spleens of PPVL rats. These similarities in gene expression profiles may suggest that mRNAs identified in human blood samples are potentially derived from the spleen.

Another study performed microarray analysis of miRNAs in splenic macrophages associated with hypersplenism due to portal hypertension in patients with hepatitis B virus (HBV)-related cirrhosis, and identified 99 differentially expressed miRNAs in splenic macrophages of portal hypertensive patients^39^. Those miRNAs included *hsa-miR-184* and *hsa-let-7i*. We found that their corresponding rat miRNAs were also differentially expressed in the spleens of PPVL rats (Table 3) and were associated with mRNAs involved in cell proliferation and innate immune responses (Figs 6 and 7, Supplementary Tables S4 and S5). In these human splenic macrophages, only one miRNA, *has-miR-615–3p*, was significantly up-regulated^39^. *has-miR-615-3p* represses expression of ligand-dependent nuclear receptor corepressor, which is a derepressor of peroxisome proliferator-activated receptor gamma^40^. In rats, no miRNA corresponding to *hsa-miR-615–3p* has been identified in the database at miRbase (http://www.mirbase.org/). Therefore, this study was not able to verify differential expression of *miR-615-3p* in the spleens of PPVL rats.

Another interesting observation in our study is that a considerable number of olfactory receptor genes involved in the *“olfactory transduction”* pathway were up- and down-regulated in the spleens of PPVL rats (Fig. 4C, Supplementary Table S3). Given that olfactory receptors may play a role in non-chemosensory tissues^41^ and that these receptors have been found outside the olfactory system including the spleen^42,43^, an identification of their ligands and signal transduction in the spleen may provide a new insight into the functional significance of olfactory receptors.

Lastly, of note is the recent direction of developing computational models for prediction of disease-associated miRNAs. Computational models are increasingly considered as an important means for identification of novel miRNA-disease associations and are expected to significantly reduce the time and cost of biological experiments^44^. For example, Chen *et al*. recently proposed several computational models for miRNA-disease associations^45–48^. One of them is the Bipartite Network Projection for miRNA-Disease Association prediction (BNPMDA) model based on the rating-integrated bipartite network recommendation and known miRNA-disease associations^46^. In this model, using agglomerative hierarchical clustering, they constructed bias ratings between miRNAs and diseases based on miRNA functional similarities and disease semantic similarities. With confirmation of its effectiveness by several validation analyses, they concluded that the BNPMDA model could be an effective computational model for prediction of potential miRNA-disease associations at a high accuracy level.

As also shown in the BNPMDA model, the development of computational models rests on experimentally verified miRNA-disease associations^44^. Currently, however, data volumes of miRNA-disease associations identified by experimental methods are limited. In addition, because there is no information available regarding the miRNA-associated regulation of mRNA expression in portal hypertension-induced splenomegaly, this study provides useful information for further prediction of miRNAs related to the pathophysiology of the spleen in portal hypertension as well as non-cirrhotic portal hypertension-related miRNAs, and thus contributes to unmet needs for accumulation of data on miRNA-disease associations.

In conclusion, we have identified a wide range of gene expression profiles, leading to unique biological consequences, such as splenic fibrosis and cell proliferation as well as potential alterations in immune responses, in PPVL-induced splenomegaly. The novel pathways discovered in this study provide the foundation for future targeted investigation into the mechanisms of splenomegaly in portal hypertension.

## Methods

### Animal experiments

All animal experiments were approved by the Institutional Animal Care and Use Committees of Yale University and the Veterans Affairs Connecticut Healthcare System and were performed in accordance with the National Institutes of Health *Guide for the Care and Use of Laboratory Animals*.

A total of six male Sprague–Dawley rats weighing 300–350 g were used. PPVL or sham surgery was performed in 3 rats each, as described previously^49^. In brief, after midline abdominal incision under anesthesia, the portal vein was separated from surrounding tissue. A 3-0 silk suture was tied around the portal vein and a 20-gauge blunt-end needle lying along it. Subsequent removal of the needle yielded a calibrated stenosis of the portal vein. In sham-operated rats, the same operation was performed, but no suture was placed. Spleens were collected ten days after surgery when portal hypertension and splenomegaly were fully established in rats with PPVL.

### Histological assessment

Formalin-fixed and paraffin-embedded spleen tissue blocks were cut into 5-μm-thick sections. Sections were stained with hematoxylin and eosin (H&E) solution for histopathological analysis and also with Sirius red to assess splenic collagen content. These histological assessments were conducted according to the methods we previously reported^50,51^. Immunostaining was performed for Ki67 (Abcam, Cambridge, MA) to determine proliferating cells and alpha-smooth muscle actin (α-SMA) (a marker of myofibroblasts and fibroblastic reticular cells) (Dako, Carpinteria, CA). Sections were visualized by light microscopy and images were acquired using an Olympus camera (Olympus, Japan) and Zeiss fluorescence microscopy (Zeiss, Germany). Analysis of digitized images was performed using ImageJ and Prism software.

### Isolation of total RNA including miRNA from spleen specimens

Total RNA including miRNA was isolated from approximately 50 mg of frozen whole spleens of PPVL and sham rats using the TRIzol reagent (Thermo Fisher Scientific, Waltham, MA) according to the manufacturer’s instructions. Purified RNA quantity and quality were determined by measuring the ratio of absorbance at 260 nm to that at 280 nm (A260/A280) using a NanoDrop 2000 Spectrophotometer (Thermo Fisher Scientific) and by on-chip capillary electrophoresis using Bioanalyzer 2100 (Agilent Technologies, Santa Clara, CA). We used the samples with the A260/A280 ratio of >1.9 and with the RNA integrity number of >9 for microarray analyses.

### Microarray hybridization

cRNA synthesis and whole genome microarray analysis were carried out at the Yale Center for Genomic Analysis (Yale University). Single-stranded cDNA was synthesized by reverse transcription using poly(A) RNA present in starting total RNA samples. Single-stranded cDNA was then converted into double-stranded cDNA and purified using Affymetrix Sample Cleanup Module (Thermo Fisher Scientific). *In vitro* transcription reaction was then carried out overnight in the presence of biotinylated UTP and CTP to produce biotin-labeled cRNA from the double-stranded cDNA. The resulting cRNA was fragmented in the presence of heat and Mg^2+^ before hybridization to the test array. Microarray analyses of mRNAs and miRNAs were performed using Affymetrix GeneChip^®^ Rat Gene 2.0 ST Array (Thermo Fisher Scientific) and GeneChip^®^ miRNA 4.0 Array (Thermo Fisher Scientific) in conjugation with the FlashTag^®^ Biotin HSR RNA Labeling Kit (Thermo Fisher Scientific), respectively. All reactions and hybridizations were carried out according to the manufacturer’s protocol. These arrays were washed with the GeneChip^®^ Fluidics Station 450 (Thermo Fisher Scientific) and scanned with the GeneChip^®^ Scanner 3000 (Thermo Fisher Scientific).

### Microarray data analysis

Differentially expressed transcripts (DETs) were analyzed with Transcriptome Analysis Console 4.0 (Thermo Fisher Scientific) (Fig. 1). The Robust Multiarray Average algorithm was used for normalization of the data to generate a single expression value for each probe set. Normalized expression values were log_2_-transformed, and empirical Bayes analysis^52^ was performed for differential expression analysis. We identified DETs using the following selection criteria: an empirical Bayes approach with a significance criterion of p < 0.05 (for mRNAs) or < 0.10 (for miRNAs) and a threshold fold change cut off value of >|1| (for mRNAs) or >|1.5| (for miRNAs) in the spleens of PPVL rats compared to those of sham rats. Our purpose was to determine a comprehensive profile of DETs in whole spleen tissues rather than specific cell populations. Therefore, more stringent selection criteria were not used to determine reproducible gene lists^53^ and to avoid limiting the datasets unnecessarily^54^. We displayed DETs in a scatter plot and heatmap with dendrogram where a distance metric was the Euclidean distance, and the distances between clusters were computed using the complete linkage method to profile gene expression patterns in the spleens of PPVL rats compared to those of sham rats.

### Functional annotation

We first converted all Affymetrix transcript cluster IDs of mRNAs to the corresponding Entrez gene IDs using DAVID version 6.8 (https://david.ncifcrf.gov/home.jsp)^55^ (Fig. 1) with the use of *Rattus norvegicus* genome as the background list for over-representation analysis. Next, we performed enriched GO analysis using the DAVID^55^ (Fig. 1) to categorize identified genes (i.e., DETs) into biological functions (specific ontology ‘terms’) constructed by biological process, molecular function and cellular component^15,16^. Significantly enriched biological processes with Benjamini’s corrected p-values of <0.05 were determined using the DAVID Functional Annotation Tool. The fold enrichment was also calculated by the ratio of the number of genes in the gene set to the expected number in the category based on the human database^55^. We also used the Cytoscape software (http://www.cytoscape.org/index.html) to visualize the results of GO analyses (Fig. 3). To determine main biological pathways in which up- or down-regulated genes are involved, pathway analysis was performed using a major public pathway-related database, KEGG (http://www.genome.jp/kegg/) (Fig. 1). In this pathway analysis, we looked into three areas of the KEGG Pathway Database, “Cellular Processes”, “Environmental Information Processing” and “Organismal Systems”, which are closely related to physiological and pathophysiological processes, and determined specific pathways that contain more than five genes up- or down-regulated in the spleens of PPVL rats.

### miRNA target prediction

Target genes for differentially expressed miRNAs were identified using miRGate (http://mirgate.bioinfo.cnio.es/miRGate/) that is composed of all gene information from EnsEMBL (http://useast.ensembl.org/index.html) and all miRNA data from miRbase (http://www.mirbase.org/) and that processes data using 5 computational tools (miRanda, RNAHybrid, Pita, microtar and Targetscan) and 4 validation tools (Mirtarbase, Mirecords, Tarbase and OncomiRDB)^56^. We used the miRGate database because it contains the most substantial number of rat miRNA sequences for prediction with a high degree of accuracy^56^. For target gene prediction, we focused on miRNAs that were differentially expressed in the spleens of PPVL rats with a fold-change of 1.5 or more. We extracted the genes which were up- and down-regulated in the spleens of PPVL rats from target genes predicted by miRGate of down- and up-regulated miRNAs, respectively. This process also revealed how much genes identified in PPVL spleens were identical with predicted target genes. Similarly, we looked into genes related to the GO terms and KEGG pathways determined as described above and verified that they were also largely predicted traget genes (Fig. 1).

## Electronic supplementary material

Supplementary Table S1

Supplementary Table S2.

Supplementary Table S3

Supplementary Table S4

Supplementary Table S5

Supplementary Figure S1

Supplementary Figure S2

## Data Availability

The datasets generated and/or analyzed in this study are available in the National Center for Biotechnology Information Gene Expression Omnibus repository with unique persistent identifiers of NCBI tracking system accession numbers: GSE113613 and GSE113612, and hyperlinks to the datasets are provided below. https://www.ncbi.nlm.nih.gov/geo/query/acc.cgi?acc=GSE113613. https://www.ncbi.nlm.nih.gov/geo/query/acc.cgi?acc=GSE113612.
