# Supplementary material for: Integrated analysis of microRNA and mRNA expression profiles in splenomegaly induced by non-cirrhotic portal hypertension in rats
Source: Sci Rep. 2018 Dec 20;8:17983. doi: 10.1038/s41598-018-36297-0 (PMC6301948; doi:10.1038/s41598-018-36297-0)
Supplement: Supplementary file 1 — Supplementary Table S1 [file 41598_2018_36297_MOESM1_ESM.docx]

**Integrated analysis of microRNA and mRNA expression profiles in splenomegaly induced by non-cirrhotic portal hypertension in rats.**

Junji Saruwatari^1,^ *, Chao Dong^1, 2,^ *, Teruo Utsumi^3^, Masatake Tanaka^1^, Matthew McConnell^1^, Yasuko Iwakiri^1, #^.

1. Section of Digestive Diseases, Yale University School of Medicine, New Haven, CT. USA
2. Department of General Surgery, Xiangya Hospital, Central South University, Changsha, China
3. VA CT Healthcare System, West Haven, CT

* Equal contributions.

**# Correspondence**

**Supplementary Table S1. List of the top three KEGG pathways involved in “Cellular Processes” sorted by the number of genes, separated by genes up- and down-regulated in the spleens of PPVL rats.**

| **KEGG No.** | **Pathway** | **Fold Change** | **Up-regulated** |  | **Down-regulated** | |
| --- | --- | --- | --- | --- | --- | --- |
|  |  |  | **KEGG term^a^:** Gene Name |  | **KEGG term^a^:** Gene Name | |
| **First pathway in up-regulated genes** | | | | | | |
| rno04510 | Focal adhesion | >2.0 | ― |  | ― | |
|  |  | >1.5 | **ECM:** Col1a1; **ITGA:** Itga8 |  | ― | |
|  |  | >1.0 | **ECM:** Col1a2, Col4a5, Thbs2, Lamb1, Lama4;  **GF:** Igf1, Figf, Vegfb; **ITGA:** Itga1; **ITGB:** Itgb5;  **Caveolin:** Cav3; **RTK:** Pdgfrb; **RhoGAP:** Arhgap35; **Parvin:** Parva; **MLCK:** Mylk; **c-Jun:** Jun; **Bcl-2:** Bcl2 |  | ― | |
| **Second pathway in up-regulated genes** | | | | | |  |
| rno04810 | Regulation of actin cytoskeleton | >2.0 | ― |  | ― | |
|  |  | >1.5 | **ITG:** Itga8; **MyosinII:** Myh10 |  | ― | |
|  |  | >1.0 | **GF:** Fgf1, Fgf2; **RTK:** Pdgfrb; **F2R/CD14:** F2r, Cd14; **ITG:** Itga1, Itgb5; **GPCR:** Bdkrb1; **Gβγ:** Gng12; **Mena:** Enah; **GRLF1:** Arhgap35; **APC:** Apc2; **MLCK:** Mylk |  | **GSN:** Scin | |
| **Third pathway in up-regulated genes** | | | | | | |
| rno04145 | Phagosome | >2.0 | ― |  | **MHC-I:** RT1-T24-3 | |
|  |  | >1.5 | **MPO:** Mpo |  | ― | |
|  |  | >1.0 | **MHC-II:** RT1-DMa, RT1-DMb, RT1-DOa, RT1-Ha; **vATPase:** Atp6v0e2; **TUBB:** Tubb6; **Collectins:** Colec12; **αVβ5:** Itgb5; **TSP:** Thbs2; **CD14:** Cd14; **DCSIGN:** Cd209b, Cd209c, Cd209d |  | **MHC-I:** RT1-M1-2, RT1-M1-4, RT1-S3 | |
| **First pathway in down-regulated genes** | | | | | | |
| rno04110 | Cell cycle | >2.0 | ― |  | ― | |
|  |  | >1.5 | ― |  | **ORC/Orc1:** Orc1 | |
|  |  | >1.0 | **TGFβ:** Tgfb3 |  | **GADD45:** Gadd45b; **Chk1, 2:** Chek2; **Mad2:** Mad2l1; **CDK2:** Cdk2; **Cdc25B,C:** Cdc25c; **HDAC:** Hdac1l; **ORC/Orc6:** Orc6; **MCM/Mcm4:** Mcm4; **Cdc7:** Cdc7 | |
| **Second pathway in down-regulated genes** | | | | | | |
| rno04218 | Cellular senescence | >2.0 | ― |  | **HLA-G:** RT1-T24-3 | |
|  |  | >1.5 | **PAI-1:** Serpine1 |  | ― | |
|  |  | >1.0 | **TGFβ:** Tgfb3 |  | **HLA-G:** RT1-M1-2, RT1-M1-4, RT1-S3; **MRE11:** Mre11a; **CHK2:** Chek2; **GADD45:** Gadd45b; **CDK2:** Cdk2 | |
| **Third pathway in down-regulated genes** | | | | | | |
| rno04217 | Necroptosis | >2.0 | ― |  | ― | |
|  |  | >1.5 | ― |  | ― | |
|  |  | >1.0 | **Bcl2:** Bcl2 |  | **TNF:** Tnf; **INF:** Ifng; **IRF9:** Irf9; **CYLD:** Cyld; **ZBP1:** Zbp1; **H2AX:** Hist3h2a; **tAIF:** Aifm1; **ESCRET-III:** RGD1566265 | |

**“^a^”** denotes the KEGG terms cited on the KEGG pathway map (http://www.genome.jp/kegg/pathway.html).

The underlined genes represent the predicted targets for up- or down-regulated miRNAs in the spleen of PPVL rats.
