# Supplementary material for: Integrated analysis of microRNA and mRNA expression profiles in splenomegaly induced by non-cirrhotic portal hypertension in rats
Source: Sci Rep. 2018 Dec 20;8:17983. doi: 10.1038/s41598-018-36297-0 (PMC6301948; doi:10.1038/s41598-018-36297-0)
Supplement: Supplementary file 2 — Supplementary Table S2. [file 41598_2018_36297_MOESM2_ESM.docx]

**Integrated analysis of microRNA and mRNA expression profiles in splenomegaly induced by non-cirrhotic portal hypertension in rats.**

Junji Saruwatari^1,^ *, Chao Dong^1, 2,^ *, Teruo Utsumi^3^, Masatake Tanaka^1^, Matthew McConnell^1^, Yasuko Iwakiri^1, #^.

1. Section of Digestive Diseases, Yale University School of Medicine, New Haven, CT. USA
2. Department of General Surgery, Xiangya Hospital, Central South University, Changsha, China
3. VA CT Healthcare System, West Haven, CT

* Equal contributions.

**# Correspondence**

**Supplementary Table S2. List of the top three KEGG pathways involved in “Environmental Information Processing” sorted by the number of genes, separated by genes up- and down-regulated in the spleens of PPVL rats.**

| **KEGG No.** | **Pathway** | **Fold Change** | **Up-regulated** |  | **Down-regulated** |
| --- | --- | --- | --- | --- | --- |
|  |  |  | **KEGG term^a^:** Gene Name |  | **KEGG term^a^:** Gene Name |
| **First pathway in up-regulated genes** | | | | | |
| rno04151 | PI3K-Akt signaling pathway | >2.0 | ― |  | ― |
|  |  | >1.5 | **ECM:** Col1a1; **ITGA:** Itga8 |  | **BRCA1:** Brca1; **PERCK:** Pck2 |
|  |  | >1.0 | **GF:** Igf1, Fgf1, Vegfb, Figf, Fgf2, Ntf3, Efna1;  **ECM:** Col1a2, Col4a5, Lamb1, Lama4, Thbs2;  **RTK:** Pdgfrb, Flt3; **CytokineR:** Ghr;  **ITGA:** Itga1; **ITGB:** Itgb5; **GPCR:** Lpar1, F2r; **Gβγ:** Gng12; **CTMP:** Them4; **GYS:** Gys1; **CREB:** Creb3l2; **Bcl-2:** Bcl2 |  | **Gβγ:** Gng5; **CDK:** Cdk2 |
| **Second pathway in up-regulated genes** | | | | | |
| rno04010 | MAPK signaling pathway | >2.0 | **c-fos:** Fos |  | ― |
|  |  | >1.5 | ― |  | ― |
|  |  | >1.0 | **GF:** Igf1, Fgf1, Fgf2, Vegfb, Figf, Ntf3, Efna1; |  | **TNF:** Tnf; **CADD45:** Cadd45b |
|  |  |  | **RTK:** Pdgfrb, Flt3; **CACN;** Cacna1c, Cacna2d1; **G12:** Gng12; **MKP:** Dusp1, Dusp7; **TGFB:** Tgfb3; **CD14:** Cd14; **MEKK2/3:** Map3k3; **c-JUN:** Jun; **MEF2C:** Mef2c |  |  |
| **Third pathway in up-regulated genes** | | | | | |
| rno04390 | Hippo signaling pathway | >2.0 | ― |  | ― |
|  |  | >1.5 | **CTGF:** Ctgf; **Pai-1:** Serpine1 |  | ― |
|  |  | >1.0 | **YAP/TAZ:** Wwtr1, **TGF-β:** Tgfb3; **BMPs:** Bmp2, Bmp6; **BMPRs:** Bmpr2; **Fzd:** Fzd1, Fzd2**; Ajub:** Ajuba; **Lats1/2:** Lats2; **TEAD:** Tead1, Tead3; **TCF/LEF:** Tcf7l1, Tcf7l2; **FGF1:** Fgf1; **APC:** Apc2 |  | ― |
| **First pathway in down-regulated genes** | | | | | |
| rno04514 | Cell adhesion molecules (CAMs) | >2.0 | ― |  | **MHC-I:** RT1-T24-3 |
|  |  | >1.5 | **NCAM:** Ncam1; **ITGA:** Itga8 |  | ― |
|  |  | >1.0 | **MHC-II:** RT1-Ha, RT1-DOa, RT1-DMb,  RT1-DMa; **B7H3:** Cd276; **SN:** Siglec1;  **CTLA4:** Ctla4; **JAM1:** F11r; **JAM3:** Jam3; **ESAM:** Esam; **SDC:** Sdc2; **MPZ:** Mpzl1 |  | **MHC-I:** RT1-M1-4, RT1-S3, RT1-M1-2; **CD8:** Cd8a; **CD226:** Cd226; **SELP:** Selp |
| **Second pathway in down-regulated genes** | | | | | |
| rno04060 | Cytokine-cytokine receptor interaction | >2.0 | ― |  | ― |
|  |  | >1.5 | ― |  | ― |
|  |  | >1.0 | **CCL2:** Ccl2; **IL6ST:** Il6st; **LIFR:** Lifr; **GHR:** Ghr; **PDGFRB:** Pdgfrb; **VEGFB:** Vegfb; **VEGFC/D:** Figf; **FLT3:** Flt3; **SF11A:** Tnfrsf11a; **TGFB3:** Tgfb3; **BMP2:** Bmp2; **BMPR2:** Bmpr2; **IL17RB:** Il17rb |  | **CCR5:** Ccr5; **IL5RA:** Il5ra; **TPO:** Thpo; **TNFSF7:** Ifng; **TNF:** Tnf; **TNFSF7:** Cd70 |
| **Third pathway in down-regulated genes** | | | | | |
| rno04630 | Jak-STAT signaling pathway | >2.0 | ― |  | ― |
|  |  | >1.5 | ― |  | ― |
|  |  | >1.0 | **Receptor:** Il6st, Pdgfrb, Ghr, Lifr; **SLIM:** Fhl1; **Bcl-2:** Bcl2 |  | **Cytokine:** Ifng; **Hormone:** Thpo; **Receptor:** Il5ra; **IRF9:** Irf9; **AOX:** Aox4 |

**“^a^”** denotes the KEGG terms cited on the KEGG pathway map (http://www.genome.jp/kegg/pathway.html).

The underlined genes represent the predicted targets for up- or down-regulated miRNAs in the spleen of PPVL rats.
