# Supplementary material for: Integrated analysis of microRNA and mRNA expression profiles in splenomegaly induced by non-cirrhotic portal hypertension in rats
Source: Sci Rep. 2018 Dec 20;8:17983. doi: 10.1038/s41598-018-36297-0 (PMC6301948; doi:10.1038/s41598-018-36297-0)
Supplement: Supplementary file 6 — Supplementary Figure S1 [file 41598_2018_36297_MOESM6_ESM.pdf]

Integrated analysis of microRNA and mRNA expression profiles in splenomegaly induced by non-cirrhotic portal hypertension in rats.

Junji Saruwatari[1, *], Chao Dong[1, 2, *], Teruo Utsumi[3], Masatake Tanaka[1], Matthew McConnell[1], Yasuko Iwakiri[1, #].

1. Section of Digestive Diseases, Yale University School of Medicine, New Haven, CT. USA
2. Department of General Surgery, Xiangya Hospital, Central South University, Changsha, China
3. VA CT Healthcare System, West Haven, CT
* Equal contributions.
# Correspondence

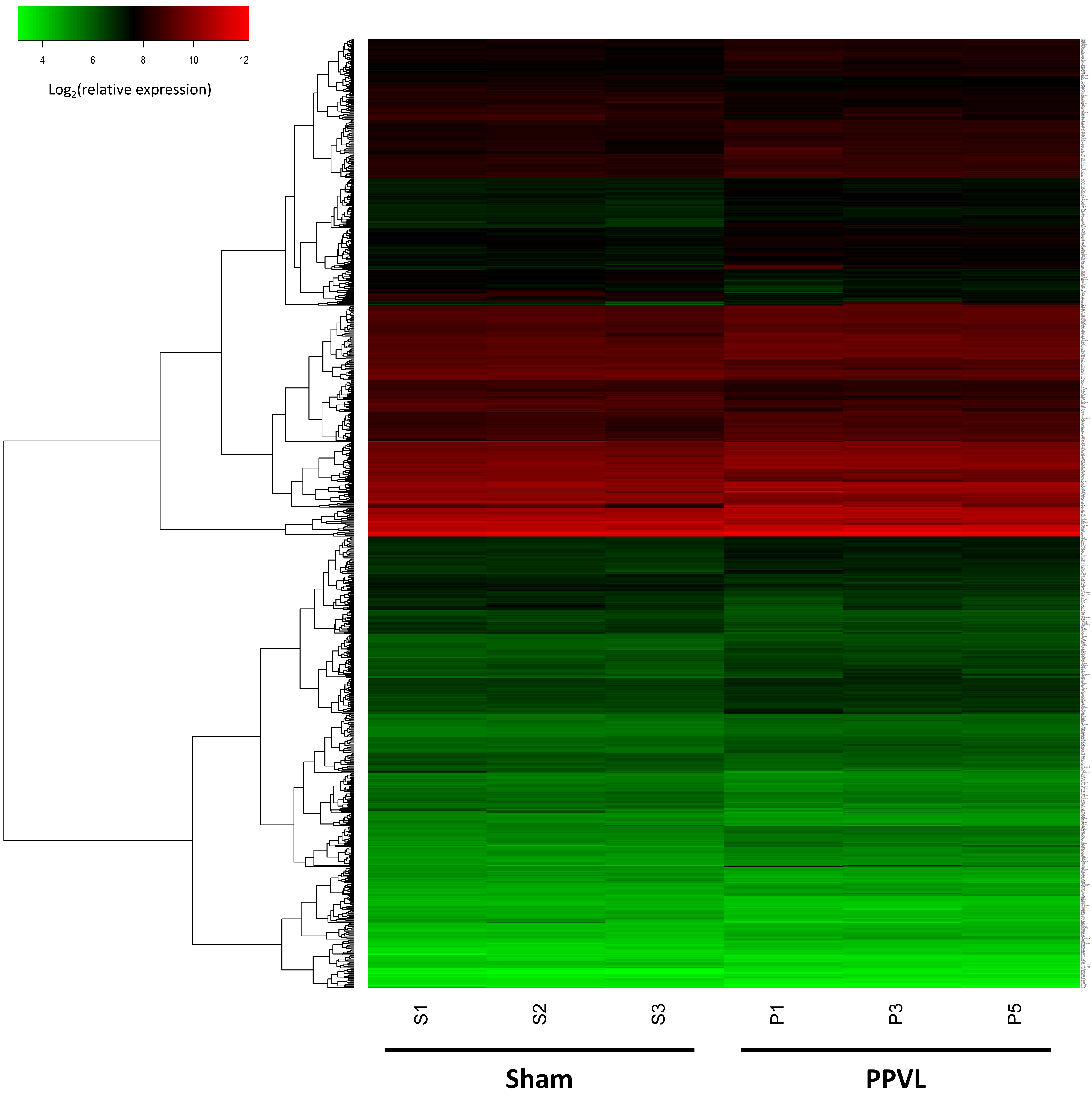

Supplementary Figure S1. Hierarchical Clustering of differentially expressed mRNAs in the spleens of PPVL rats compared to those of sham rats.
