# Supplementary material for: Integrated analysis of microRNA and mRNA expression profiles in splenomegaly induced by non-cirrhotic portal hypertension in rats
Source: Sci Rep. 2018 Dec 20;8:17983. doi: 10.1038/s41598-018-36297-0 (PMC6301948; doi:10.1038/s41598-018-36297-0)
Supplement: Supplementary file 7 — Supplementary Figure S2 [file 41598_2018_36297_MOESM7_ESM.pdf]

Integrated analysis of microRNA and mRNA expression profiles in splenomegaly induced by non-cirrhotic portal hypertension in rats.

Junji Saruwatari[1, *], Chao Dong[1, 2, *], Teruo Utsumi[3], Masatake Tanaka[1], Matthew McConnell[1], Yasuko Iwakiri[1, #].

1. Section of Digestive Diseases, Yale University School of Medicine, New Haven, CT. USA
2. Department of General Surgery, Xiangya Hospital, Central South University, Changsha, China
3. VA CT Healthcare System, West Haven, CT
* Equal contributions.
# Correspondence

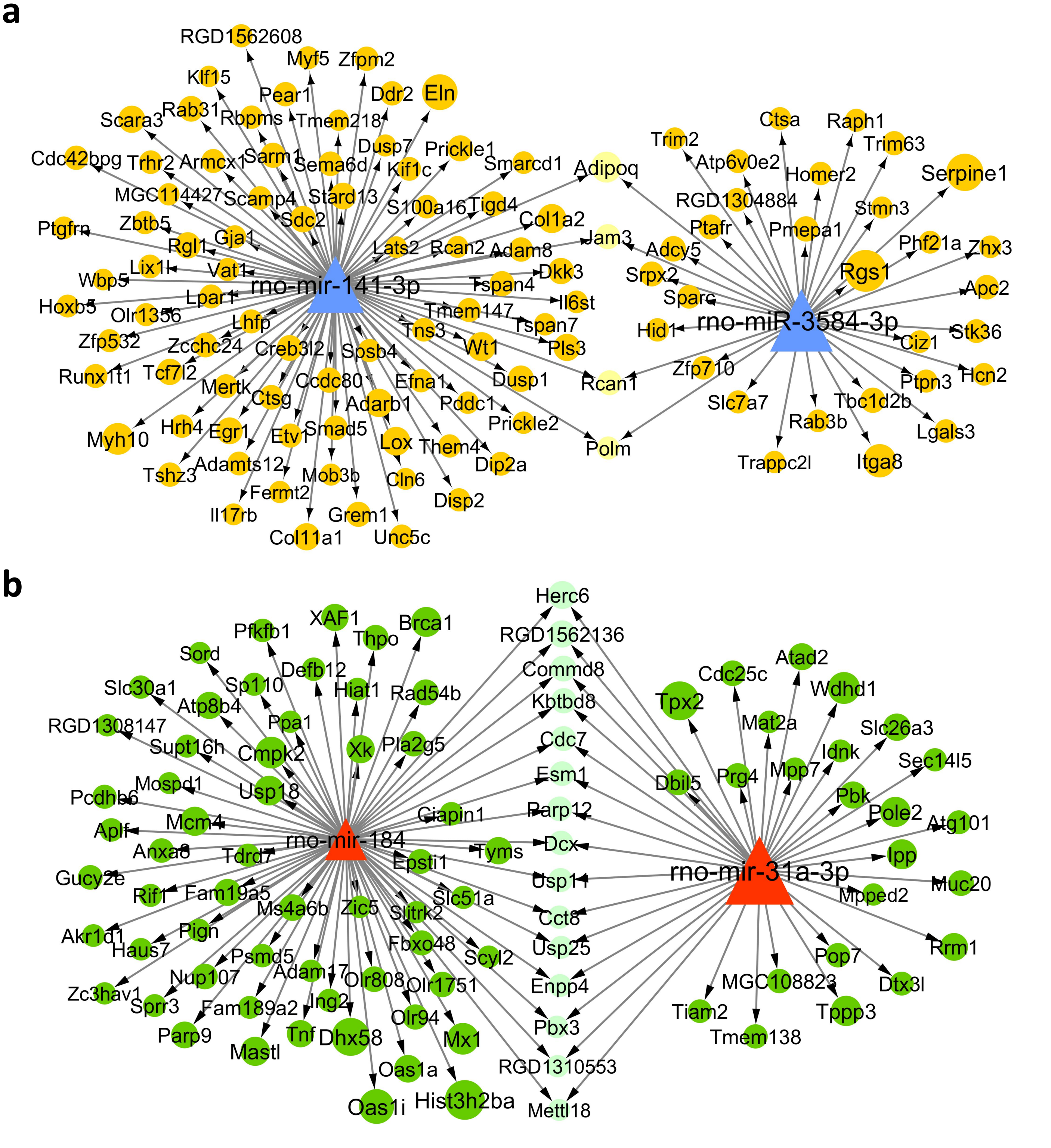

Supplementary Figure S2. The miRNA-mRNA network analysis in the spleen.
(**a**) The network consists of the two miRNAs >2.0-fold down-regulated, rno-miR-141-3p and rno-miR-3584-3p (blue triangles); their target genes (yellow) and the overlapped target genes (light yellow) up-regulated in the spleens of PPVL. (**b**) The network consists of the two miRNAs >2.0-fold up-regulated, rno-miR-184 and rno-miR-31a-3p (red triangles); their target genes (green) and the overlapped target genes (light green) down-regulated in the spleens of PPVL rats compared to those from sham rats.
The sizes of the triangles and circles represent the differences in the fold-change in the spleens from PPVL rats compared to those from sham rats.
